# Functional Wet Noodles Based on Modified Cassava Flour and Pumpkin Flour: Physicochemical, Bioactive, Structural, and Starch Digestibility Characteristics

**DOI:** 10.1155/ijfo/7244914

**Published:** 2026-08-21

**Authors:** Agus Slamet, Wafit Dinarto, Sundari Sundari

**Affiliations:** ^1^ Department of Agricultural Product Technology, Faculty of Agroindustry, Universitas Mercu Buana Yogyakarta, Yogyakarta, Indonesia, mercubuana-yogya.ac.id; ^2^ Department of Agrotechnology, Faculty of Agroindustry, Universitas Mercu Buana Yogyakarta, Yogyakarta, Indonesia, mercubuana-yogya.ac.id; ^3^ Department of Animal Husbandry, Faculty of Agroindustry, Universitas Mercu Buana Yogyakarta, Yogyakarta, Indonesia, mercubuana-yogya.ac.id

**Keywords:** functional wet noodles, mocaf, modified cassava flour, pumpkin flour, resistant starch, starch digestibility

## Abstract

The use of local food ingredients in noodle products is increasing due to their potential to improve nutritional value and functional properties. This study evaluated the effect of partially substituting wheat flour with modified cassava flour (mocaf) and pumpkin flour on the physicochemical characteristics, bioactive compound content, starch digestibility fractions, structural properties, and sensory acceptance of fresh noodles. Noodles were prepared using five wheat flour: mocaf: pumpkin flour ratios of 100:0:0, 80:10:10, 70:20:10, 60:20:20, and 50:25:25 (*w*/*w*). Physicochemical properties, bioactive compounds, starch digestibility fractions, and structural characteristics were evaluated through color measurements, texture analysis (hardness and deformation), cooking quality assessment, *β*‐carotene and total phenolic determination, antioxidant activity analysis, in vitro starch digestibility evaluation, Fourier‐transform infrared spectroscopy (FTIR), and scanning electron microscopy (SEM). Increasing the proportion of mocaf and pumpkin flour significantly (*p* < 0.05) reduced noodle hardness from 7.79 to 5.35 N and deformation from 11.35% to 6.03%, while increasing cooking loss from 8.24% to 12.75%. Substitution also significantly increased *β*‐carotene (2.29–124.46 *μ*g/g) and total phenolic contents (0.14–4.94 mg GAE/g) (*p* < 0.05). The in vitro starch digestibility results indicated lower levels of RDS and an estimated glycemic index, whereas RS content increased. The 60:20:20 formulation exhibited the highest sensory acceptance. These results indicate that mocaf and pumpkin flour substitution improves the functional and nutritional properties of fresh noodles and supports their development as functional foods based on local ingredients.

## 1. Introduction

Food diversification based on local resources is a key strategy for supporting food security and developing functional foods, particularly in tropical countries with a wide variety of nonwheat food commodities. The development of widely consumed food products using local raw materials with enhanced nutritional quality is gaining increasing attention within sustainable food system approaches [1]. Noodles are cereal‐based foods widely consumed across Asia and other regions worldwide [2]. Noodle consumption continues to rise as people′s lifestyles shift toward more convenient foods that require relatively little preparation. Alongside convenience, greater attention is now directed by modern consumers toward health considerations, nutrient composition, and bioactive constituents in their food choices [3]. Therefore, the development of noodle products focuses not only on sensory characteristics and physical quality, but also on enhancing their nutritional value and health‐related functional benefits.

Most conventional noodle products still use wheat flour as their main ingredient. Wheat flour can form gluten networks, which give the dough its elastic structure [4]. However, wheat production in many tropical countries is relatively limited, so the demand for wheat flour is still largely met through imports. Dependence on imported wheat can increase the food system′s vulnerability and raise food production costs [5]. These conditions underscore the need to develop noodle products made from local ingredients as part of a strategy to diversify the food sector and strengthen food self‐reliance. Utilizing local carbohydrate sources can also increase the value of local agricultural commodities and expand innovation in food products based on domestic resources [6].

One local ingredient with potential as a wheat‐flour substitute is modified cassava flour (mocaf) [7]. Mocaf is a cassava‐based flour obtained through fermentation, a process that alters its physicochemical properties, particularly the structure of starch granules and their hydration behavior [8]. This fermentation treatment increases water absorption capacity, enhances starch functional properties, and produces dough with greater stability compared with conventional cassava flour. Furthermore, cassava is widely cultivated in tropical regions, so its use as a food ingredient can add value to the development of products based on local resources. Several studies have reported that mocaf can be used as a substitute ingredient in various flour‐based food products, including noodle products [9, 10]. However, the absence of gluten protein in mocaf can affect dough structure and potentially compromise the textural characteristics of the noodle product if the formulation is not properly optimized.

In addition to using local ingredients as substitutes for wheat flour, the functional value of noodle products can also be enhanced by adding food ingredients rich in bioactive compounds. Pumpkin (*Cucurbita moschata* Duchesne) is a horticultural crop widely cultivated in various Asian countries and is known to contain relatively high levels of *β*‐carotene, phenolic compounds, vitamins, and dietary fiber [11]. Pumpkin is recognized for its health‐promoting properties, attributed to its rich content of carotenoids, tocopherols, and antioxidant compounds [12]. As a provitamin A, *β*‐carotene exhibits antioxidant activity that helps protect cells against oxidative stress induced by free radicals [13]. Phenolic compounds present in pumpkin have been shown to exhibit antioxidant activity, thereby potentially increasing the functional value of food products [14]. According to Slamet et al. [15], an instant porridge formulated from a combination of brown rice and pumpkin was able to restore pancreatic cell damage in diabetic mice. Several other studies have shown that adding pumpkin flour to noodle products can enhance their natural color, antioxidant activity, and nutritional value [16]. However, the fiber and pectin present in pumpkin may influence gluten network development and consequently modify the textural properties of the noodle product.

Starch digestibility is an essential consideration in the development of starch‐based noodles, alongside ingredient composition and bioactive compounds. This digestibility is typically described using starch fractions, including rapidly digestible starch (RDS), slowly digestible starch (SDS), and resistant starch (RS), which reflect starch behavior in the human digestive system [17]. The proportions of these starch fractions are closely related to the glycemic response of the food, which can be expressed as the estimated glycemic index (eGI) [18]. Food products containing higher proportions of resistant and SDS are typically associated with reduced glycemic responses and may offer benefits as healthier functional foods [19]. Furthermore, alterations in molecular and microstructural features contribute to determining the characteristics of the noodle product [20]. The incorporation of gluten‐free ingredients such as mocaf, together with dietary fiber from pumpkin, may influence starch–protein interactions within the dough matrix [21]. Structural analysis techniques, such as Fourier transform infrared (FTIR) and scanning electron microscopy (SEM), are used to elucidate molecular and microstructural changes arising from compositional modifications in starch‐based products [22]. These structural analyses offer insights into how modifications in starch structure are associated with the functional properties of food products [23].

However, research integrating the substitution of mocaf and pumpkin flour, particularly in relation to their effects on starch structural characteristics and digestibility in noodle products, is still limited. Unlike previous studies that primarily focused on physicochemical quality, sensory characteristics, or nutritional composition, this study provides an integrated evaluation of fresh noodles formulated with mocaf and pumpkin flour by simultaneously examining physicochemical properties, bioactive compounds, starch digestibility fractions (RDS, SDS, and RS), eGI, and structural characteristics at both molecular (FTIR) and microstructural (SEM) levels. Furthermore, the findings provide practical insights for the development of nutritionally enhanced fresh noodles utilizing locally available ingredients, thereby supporting food diversification strategies and the potential industrial application of mocaf and pumpkin flour in value‐added noodle products. The present study investigates the impact of substituting wheat flour with mocaf and pumpkin flour on the physicochemical properties, chemical composition, bioactive compounds, starch fractions, eGI, structural characteristics, and sensory acceptance of fresh noodles.

## 2. Materials and Methods

### 2.1. Materials

The raw materials included mocaf (Mocafine) and pumpkin flour (Nula), which were obtained from local industries in Sleman, Yogyakarta, Indonesia, whereas high‐protein wheat flour was purchased from local markets in Yogyakarta. The mocaf flour has a moisture content of less than 12% and a particle size of approximately 80 mesh. The pumpkin flour used has a particle size that passes an 80‐mesh sieve with a moisture content of less than 10%. The food additives used include sodium chloride (NaCl) and sodium tripolyphosphate (STPP; Na_5_P_3_O_10_).

All chemicals of analytical grade were supplied by Merck (Darmstadt, Germany) and Sigma‐Aldrich (St. Louis, United States). The reagents comprised petroleum ether, sulfuric acid (H_2_SO_4_), sodium hydroxide (NaOH), boric acid (H_3_BO_3_), hydrochloric acid (HCl), ethanol, n‐hexane, acetone, methanol (HPLC grade), Folin–Ciocalteu reagent, sodium carbonate (Na_2_CO_3_), gallic acid standard, and DPPH. Starch fraction analysis employed *α*‐amylase and glucoamylase, while sodium acetate buffer and glucose standards were used for starch hydrolysis. All reagents were used as received without any further purification.

### 2.2. Methods

#### 2.2.1. Making Wet Noodles

The fresh noodles were formulated using five different ratios of wheat flour: mocaf: pumpkin flour, namely 100:0:0, 80:10:10, 70:20:10, 60:20:20, and 50:25:25 (*w*/*w*). A uniform blend of wheat flour, mocaf, and pumpkin flour was obtained, followed by the addition of salt (1.5%, *w*/*w*) and STPP (0.3%, *w*/*w*), which was maintained at the same level in all formulations. Next, water was added gradually while kneading for about 10 min until a homogeneous, elastic dough formed.

The dough was then rested for 30 min at room temperature (27^°^C ± 2^°^C) to allow for the hydration of the starch and protein components. After hydration, the dough was rolled using a pasta machine to produce a dough sheet approximately 1.5 mm thick, then cut into noodle strands approximately 2 mm wide. The resulting noodles were boiled in boiling water at a water‐to‐sample ratio of 10:1 (*v*/*w*) for 3 min. Once boiled, the noodles were drained and cooled at ambient temperature for 10 min before storage at 4^°^C ± 1^°^C until analysis.

#### 2.2.2. Proximate Analysis

The proximate composition of uncooked fresh noodles was determined according to AOAC International standards [24]. Moisture was measured by oven drying (Method 925.10), ash by combustion in a muffle furnace (Method 923.03), crude fat by Soxhlet extraction (Method 989.05), and crude protein using the Kjeldahl method (Method 991.20). Carbohydrate content was subsequently calculated by difference using the following equation:

(1) Carbohydrate %=100−moisture+ash+fat+protein.

#### 2.2.3. Color Analysis

Color of uncooked fresh noodles was determined using a colorimeter (Konica Minolta CR‐400, Osaka, Japan) according to the CIE L*a*b* system, including lightness (L*), redness–greenness (a*), and yellowness–blueness (b*). Measurements were performed at three points per sample and reported as the average.

#### 2.2.4. Texture Analysis

The hardness and deformation properties of the cooked noodles were determined using a texture analyzer (TA.XT Plus, Stable Micro Systems, United Kingdom). Samples were compressed to 50% of their initial height at a test speed of 1 mm/s, following a previously reported method Li et al. [25]. The parameters evaluated included hardness and deformation.

#### 2.2.5. Cooking Loss

Cooking loss was determined gravimetrically using cooked noodle samples by boiling 25 g of noodle samples in 250 mL of water for 3 min, followed by filtration of the cooking water to remove insoluble residues. After filtration, the filtrate was dried at 105°C in an oven until a constant weight was obtained, and the loss was calculated as the percentage of solids released during cooking based on the initial sample weight.

#### 2.2.6. Analysis of *β*‐Carotene

*β*‐Carotene content was determined according to the method described by Mohammed et al. [26] with slight modifications. Briefly, 5 g of dried noodle sample was extracted with 25 mL of an n‐hexane:acetone:ethanol mixture (2:1:1, *v*/*v*/*v*). The mixture was vortexed for 2 min and centrifuged at 4000 rpm for 10 min. The resulting supernatant was filtered through Whatman No. One filter paper prior to analysis. Absorbance was measured at 450 nm using a UV–Vis spectrophotometer (Shimadzu UV‐1800, Japan). *β*‐Carotene concentration was quantified using a calibration curve prepared from analytical‐grade *β*‐carotene standards and expressed as *μ*g/g dry matter.

#### 2.2.7. Analysis of Total Phenolic Content (TPC)

TPC of uncooked wet noodle samples was determined using the Folin–Ciocalteu method described by Singleton et al. [27]. Briefly, 0.5 mL of the sample extract was mixed with 2.5 mL of 10% Folin–Ciocalteu reagent and 2 mL of 7.5% sodium carbonate solution. The reaction mixture was incubated at room temperature for 30 min to allow color development. Absorbance was then measured at 765 nm using a UV–Vis spectrophotometer. TPC was quantified using a gallic acid calibration curve and expressed as mg gallic acid equivalents per g sample (mg GAE/g).

#### 2.2.8. Antioxidant Activity (DPPH)

Antioxidant activity of uncooked wet noodle samples was assessed using the DPPH free radical scavenging assay following the procedure of Brand‐Williams et al. [28] For the analysis, 1 mL of the sample extract (1 mg/mL), prepared as described previously, was added to 3 mL of 0.1‐mM DPPH solution in methanol. The mixture was then kept under dark conditions at room temperature for 30 min to allow the reaction to proceed. Subsequently, absorbance was measured at 517 nm using a UV–Vis spectrophotometer. A control solution consisting of 3‐mL DPPH solution and 1‐mL methanol was used as the blank. The free radical scavenging activity was expressed as a percentage and calculated using the following equation:

(2) Radical scavenging activity %=1−AT/Ao×100%.

#### 2.2.9. Characterization of Starch Fractions and Glycemic Index

The determination of starch fractions was carried out using uncooked fresh noodle samples and a validated in vitro enzymatic hydrolysis protocol adapted from Li et al. [29], which simulates physiological starch digestion conditions through sequential hydrolysis with *α*‐amylase and glucoamylase. Samples were dried, finely ground, and weighed (100 mg) before incubation with sodium acetate buffer (pH 5.2) containing *α*‐amylase and glucoamylase at 37°C in a shaking water bath. Aliquots were withdrawn at defined time intervals (20 and 120 min), and enzymatic activity was terminated using absolute ethanol. The glucose released was quantified spectrophotometrically and used to estimate starch fractions. The classification of starch fractions was based on hydrolysis duration: RDS (within 20 min), SDS (20–120 min), and RS (remaining after 120 min). The starch fractions are calculated using the following equations:

(3) RDS %=G200.9×,

(4) SDS %=G12020−G×0.9,

(5) RS %=Total starch−RDS+SDS,

where G20 = glucose released at 20 min of hydrolysis; G120 = glucose released at 120 min of hydrolysis; and 0.9 = conversion factor from glucose to starch equivalent.

A starch hydrolysis profile was constructed by relating the amount of glucose released to incubation time. The AUC of the sample was subsequently compared with that of white bread as the reference standard to obtain the hydrolysis index (HI). The HI value was calculated using the following equation:

(6) HI%=AUC sample/AUC reference×100,

where AUC_sample = sample hydrolysis curve area, and AUC_reference = reference hydrolysis curve area (white bread or glucose).

The eGI is calculated from the HI using the empirical equation developed by Goñi et al. [30] with minor modifications

(7) eGI=39.71+0.549×HI.

The eGI value is used to estimate the potential glycemic response of the produced fresh noodle products.

#### 2.2.10. Structural Analysis (FTIR and SEM)

The molecular structure of uncooked fresh noodles was analyzed by FTIR spectroscopy (Bruker Tensor 27, Germany) in the range of 4000–400 cm^−1^ with a spectral resolution of 4 cm^−1^ to identify functional groups and potential starch–protein interactions. Microstructural characteristics were evaluated using SEM (Hitachi SU3500, Japan) at magnifications between 500× and 5000×. Samples were dried at 40°C for 24 h and gold‐coated to improve conductivity before analysis.

#### 2.2.11. Sensory Test

A sensory preference test was conducted to assess panelists′ acceptance of the formulated cooked wet noodles. Sensory evaluation was conducted with 30 semitrained assessors recruited from undergraduate students of the Food Technology Study Program at Universitas Mercu Buana Yogyakarta, Indonesia. Participants were 18–25 years of age and reported having no known allergies to the ingredients used in the evaluated samples. The attributes of color, aroma, texture, and overall acceptance were assessed using a 5‐point hedonic rating system, where a score of 1 indicated *strong dislike* and a score of 5 indicated *strong liking*. In a sensory laboratory maintained at 22^°^C ± 2^°^C with neutral white lighting, samples were served at 45^°^C ± 3^°^C, labeled with three‐digit random codes, and presented in randomized order. Panelists rinsed their mouths with mineral water between samples to minimize carry‐over effects.

#### 2.2.12. Research Design and Statistical Analysis

A single‐factor completely randomized design (CRD) was employed, in which the treatment variable consisted of different formulations of wheat flour, mocaf, and pumpkin flour at proportions of 100:0:0, 80:10:10, 70:20:10, 60:20:20, and 50:25:25. Each formulation was prepared in triplicate as independent production batches (biological replicates), and all analytical measurements were subsequently performed in triplicate for each batch (analytical replicates). The assumptions of normality and homogeneity of variance were verified prior to statistical analysis. Data were analyzed by one‐way ANOVA (*p* < 0.05) followed by Duncan′s Multiple Range Test (DMRT) using IBM SPSS Statistics for Windows, Version 25.0 (IBM Corp., Armonk, New York, United States).

## 3. Results and Discussion

### 3.1. Chemical Composition

The proximate composition of wet noodles, including moisture, ash, fat, protein, and carbohydrate contents, varied significantly with changes in the formulation ratio of wheat flour, mocaf, and pumpkin flour (Table 1).

**Table 1:** Proximate composition of wet noodle formulations.

Wheat flour: mocaf: pumpkin flour (*w*/*w*)	Moisture (%)	Ash (%)	Fat (%)	Protein (%)	Carbohydrates (%)
100:0:0	31.35 ± 0.18^d^	1.09 ± 0.25^e^	0.66 ± 0.05^c^	12.78 ± 1.37^a^	54.12 ± 1.09^a^
80:10:10	33.18 ± 0.11^c^	1.35 ± 0.14^d^	0.72 ± 0.09^b^	9.64 ± 1.34^b^	55.11 ± 2.54^a^
70:20:10	35.65 ± 0.80^b^	2.66 ± 0.15^c^	1.02 ± 0.14^a^	8.84 ± 0.69^c^	51.63 ± 1.30^c^
60:20:20	35.47 ± 0.19^b^	2.95 ± 0.27^b^	1.04 ± 0.09^a^	7.58 ± 0.74^d^	52.96 ± 2.91^b^
50:25:25	36.30 ± 0.07^a^	3.20 ± 0.17^a^	1.02 ± 0.15^a^	6.85 ± 1.57^e^	52.63 ± 1.74^b^

*Note:* Data are reported as mean ± SD, and statistical differences within a column are indicated by distinct superscript letters (*p* < 0.05).

Table 1 shows that the moisture level of wet noodles rose significantly, increasing from 31.35% to 36.30% (*p* < 0.05). This increase can be attributed to the greater water‐binding capacity of amorphous starch and dietary fiber present in mocaf and pumpkin flour compared with wheat flour. Pumpkin flour contains dietary fiber and pectin that have been reported to enhance water retention [31] and water‐binding capacity in food systems [32].

The ash content showed a significant increase (*p* < 0.05), from 1.09% in control samples to 3.20% with higher incorporation of mocaf and pumpkin flour. This increase may be associated with the mineral composition of pumpkin flour reported in previous studies, including potassium, magnesium, and phosphorus [33]. These findings suggest that incorporating local ingredients can enhance the mineral content of wet noodles.

The fat content increased from 0.66% to around 1.02%–1.04% (*p* < 0.05) in formulations with a higher proportion of pumpkin flour [34]. The fat content increased from 0.66% to approximately 1.02%–1.04% as the proportion of pumpkin flour increased. This result suggests that pumpkin flour contributed to the lipid fraction of the noodle formulations.

Protein content decreased significantly (*p* < 0.05) with increasing proportions of mocaf and pumpkin flour, declining from 12.78% in the control (100:0:0) to 6.85% in the 50:25:25 formulation. This decrease was attributed to a reduction in gluten in the noodle dough [34]. Gluten is reduced when wheat flour is replaced with mocaf and pumpkin flour, both of which do not contain gluten. Previous studies have reported that mocaf generally contains lower protein levels than wheat flour, whereas pumpkin flour contributes dietary fiber rather than gluten‐forming proteins, which may contribute to the reduction in protein content observed in the noodle formulations.

The carbohydrate content ranges from 51% to 55%, with a slight decrease observed in formulations with the highest substitution. This is because an increase in water and ash content will proportionally reduce the carbohydrate content. Pumpkin contains both soluble and insoluble fibers, whose structure differs from that of wheat carbohydrates, which are primarily composed of starch. Mocaf has starch that binds water more easily, resulting in a decrease in the carbohydrate fraction [35].

### 3.2. Color

Changes in the color attributes of wet noodles, represented by L*, a*, and b* values, were consistently observed with increasing proportions of mocaf and pumpkin flour (Table 2). The L* value decreased significantly from 81.35 in the control to 72.78 in the 50:25:25 formulation, indicating a reduction in product brightness. This decrease reflects the dominance of carotenoid pigments derived from pumpkin, which absorb blue–green light, resulting in a darker color. Additionally, increasing the proportion of pumpkin flour contributed to a more intense yellow–orange color in the final product due to its carotenoid content. During cooking, interactions among starch, protein, and bioactive compounds can influence the product′s optical properties, leading to a darker color. A decrease in lightness (L*) following pumpkin flour incorporation into food products has been reported by Slamet et al. [36]. This reduction is mainly attributed to carotenoid pigments present in pumpkin flour, which impart a yellow–orange color and consequently decrease product brightness. In addition, the use of RS in noodle products has been reported to reduce lightness [37]. This effect may be associated with changes in starch matrix structure that alter light scattering properties within the product.

**Table 2:** Color characteristics of wet noodles.

Wheat flour: mocaf: pumpkin flour (*w*/*w*)	L*	a*	b*
100:0:0	81.35 ± 3.77^a^	2.08 ± 0.53^d^	11.98 ± 1.01^d^
80:10:10	79.82 ± 4.24^b^	4.47 ± 0.40^c^	17.03 ± 1.89^c^
70:20:10	76.28 ± 3.46^c^	5.38 ± 0.75^b^	28.78 ± 2.75^b^
60:20:20	73.82 ± 4.60^d^	6.59 ± 0.58^a^	32.64 ± 2.68^a^
50:25:25	72.78 ± 3.53^e^	6.75 ± 0.62^a^	33.59 ± 2.70^a^

*Note:* Data are reported as mean ± SD, and statistical differences within a column are indicated by distinct superscript letters (*p* < 0.05).

As the level of pumpkin flour increased, the a* and b* values of fresh noodles exhibited a consistent upward trend. The a* parameter increased from 2.08 in the control to 6.75 in the formulation containing a 50:25:25 ratio. The increase in the b* value from 11.98 to 33.59 indicates a stronger yellow–orange color intensity due to the *β*‐carotene content in the pumpkin flour. This trend indicates a positive relationship between carotenoid content and the product′s yellowness. Carotenoids are known to play a direct role in imparting the characteristic yellow color of pumpkin and are relatively stable under the processing conditions typically applied during fresh noodle production, which generally involve dough mixing and short cooking periods at temperatures close to 100°C. This compound contributes to the yellow–orange color in various pumpkin‐based food products [38]. In addition, the distribution of carotenoid pigments within the noodle dough matrix can influence the color intensity of the final product. During cooking, carotenoid pigments can disperse within the starch and protein matrix, thereby enhancing the yellow–orange color intensity of the resulting fresh noodles. This indicates that increasing the proportion of pumpkin flour not only boosts the content of bioactive compounds but also improves the product′s color characteristics, resulting in a more appealing appearance for consumers.

**Figure 1:**
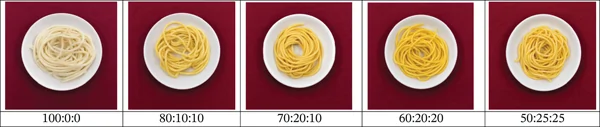
Appearance of wet noodles formulated with different wheat flour: mocaf: pumpkin flour ratios.

A progressive change in noodle color from pale yellow to deep orange–yellow was observed, as depicted in Figure 1. The 60:20:20 formulation produced the most uniform color, while the 50:25:25 formulation appeared darker and duller due to a decrease in L* value. This color change is beneficial because it gives functional noodles a distinctive visual characteristic without the addition of synthetic coloring. The orange–yellow color is produced by pumpkin carotenoid compounds [39].

### 3.3. Texture, Deformation, and Cooking Loss

Significant differences in hardness, deformation, and cooking loss were observed among wet noodle formulations prepared with varying proportions of wheat flour, mocaf, and pumpkin flour, as shown in Table 3.

**Table 3:** Hardness, deformation, and cooking loss of wet noodles.

Wheat flour: mocaf: pumpkin flour (*w*/*w*)	Hardness (N)	Deformation (%)	Cooking loss (%)
100:0:0	7.79 ± 0.79^a^	11.35 ± 1.54^a^	8.24 ± 1.01^e^
80:10:10	7.18 ± 1.05^b^	10.60 ± 1.80^b^	9.87 ± 0.97^d^
70:20:10	6.35 ± 1.87^c^	8.86 ± 1.43^c^	10.35 ± 1.53^c^
60:20:20	5.57 ± 1.41^d^	8.75 ± 1.74^c^	11.66 ± 1.49^b^
50:25:25	5.35 ± 0.97^e^	6.03 ± 1.73^d^	12.75 ± 1.63^a^

*Note:* Data are reported as mean ± SD, and statistical differences within a column are indicated by distinct superscript letters (*p* < 0.05).

A significant reduction in fresh noodle hardness was observed, with values decreasing from 7.79 N in the control formulation to 5.35 N in the 50:25:25 formulation (Table 3). This decline was primarily associated with the reduced gluten content resulting from the substitution of a portion of the wheat flour with mocaf and pumpkin flour. Gluten plays a crucial role in forming an elastic, continuous protein network in noodle dough; thus, a reduction in gluten can weaken the protein–starch matrix, resulting in a softer noodle texture. Additionally, pumpkin flour may contribute to increased water retention within the dough matrix, which could weaken the protein–starch network and reduce noodle hardness. The incorporation of nonwheat ingredients into dough formulations has been reported to decrease noodle hardness by modifying the gluten network structure [40].

The deformation of wet noodles also decreased from 11.35% in the control formulation to 6.03% in the formulation with the highest substitution level. This decrease indicates a reduced ability of the dough structure to return to its original shape after being subjected to pressure. The reduction may be attributed to the partial replacement of wheat flour with mocaf and pumpkin flour, which weakens the gluten network responsible for maintaining the elasticity and structural integrity of the noodle matrix. This condition is characterized by weakened interactions between gluten chains, including reduced formation of disulfide bonds that help maintain the elasticity of the protein network. Additionally, new interactions between starch and protein via hydrogen bonding can modify the dough matrix structure, thereby affecting its elastic properties [41]. This phenomenon is common in food systems where the proportion of nongluten ingredients has increased.

Cooking loss increased from 8.24% in the control formulation to 12.75% in the 50:25:25 formulation. The high cooking loss value indicates weakening of the starch and protein matrix, leading to some of the solid components dissolving readily into the water during boiling [42]. The reduced gluten network and the incorporation of pumpkin flour may facilitate water penetration into the dough matrix, thereby promoting the release of soluble solids during cooking. Several studies have also reported that substituting wheat flour with nonwheat ingredients tends to increase cooking loss in noodle products [43]. This effect is generally attributed to the disruption of the gluten network, which reduces the ability of the dough matrix to retain starch granules and other soluble components during cooking. Nevertheless, the 60:20:20 formulation still yields cooking loss values within an acceptable range for fresh noodles, indicating that the ingredient balance in this formulation can maintain the product′s structural integrity. Overall, the findings suggest that increasing the proportion of mocaf and pumpkin flour leads to a softer noodle texture and increased cooking loss. However, the formulation with a moderate substitution level (60:20:20) still maintains relatively good structural integrity. This indicates that this formulation can balance textural characteristics and structural stability during cooking, making it a potentially optimal formulation for developing wet noodles from local ingredients.

### 3.4. Bioactive Compounds and Antioxidant Activity

The incorporation of higher amounts of pumpkin flour resulted in a significant enhancement of *β*‐carotene content in wet noodles (*p* < 0.05) (Table 4).

**Table 4:** Bioactive components of wet noodles: *β*‐carotene, total phenolics, and antioxidant activity.

Wheat flour: mocaf: pumpkin flour (*w*/*w*)	ß‐carotene (*μ*g/g)	Total phenolic content (mg GAE/g)	Antioxidant activity (%)
100:0:0	2.29 ± 0.82^e^	0.14 ± 0.11^e^	2.69 ± 0.84^e^
80:10:10	37.68 ± 3.61^d^	2.36 ± 0.34^d^	18.47 ± 2.42^d^
70:20:10	75.53 ± 5.87^c^	3.43 ± 0.41^c^	29.54 ± 3.58^c^
60:20:20	105.57 ± 6.70^b^	3.92 ± 0.86^b^	37.71 ± 3.76^b^
50:25:25	124.46 ± 6.95^a^	4.94 ± 1.58^a^	49.75 ± 3.04^a^

*Note:* Data are reported as mean ± SD, and statistical differences within a column are indicated by distinct superscript letters (*p* < 0.05).

The *β*‐carotene content increased from 2.29 *μ*g/g in the control formulation (100:0:0) to 124.46 *μ*g/g in the formulation with a 50:25:25 ratio. The increase in *β*‐carotene content may be attributed to the incorporation of pumpkin flour, which has been reported as a rich source of carotenoids, particularly *β*‐carotene [44]. *β*‐Carotene is a carotenoid pigment that gives food its yellow to orange color. In addition, this compound exhibits biological activity as a provitamin A and acts as an antioxidant [45]. The incorporation of increasing amounts of pumpkin flour contributed to a greater presence of carotenoid pigments in the noodle matrix, thereby elevating *β*‐carotene content in the final product. These results indicate that partial substitution of wheat flour with pumpkin flour enhances bioactive compounds in fresh noodles, offering potential nutritional benefits and supporting the utilization of local ingredients in functional food development. In addition to *β*‐carotene, total phenolic compounds also contributed to the bioactive profile of the wet noodles, as evidenced by the significant increase in TPC from 0.14 to 4.94 mg GAE/g with increasing levels of mocaf and pumpkin flour substitution.

The incorporation of increasing proportions of mocaf and pumpkin flour resulted in a significant elevation of TPC (*p* < 0.05). The values increased from 0.14 mg GAE/g in the control formulation to 4.94 mg GAE/g in the 50:25:25 treatment. This enhancement reflects the contribution of substitute ingredients to the enrichment of phenolic compounds in the product matrix. Phenolic compounds are recognized as important secondary metabolites commonly found in plant‐based foods [46]. The antioxidant activity of these compounds is attributed to their capacity to neutralize free radicals by donating electrons or hydrogen atoms [47]. Within the wet noodle matrix, phenolic compounds can interact with starch and protein, which may influence their stability and biological activity during processing [48]. The increase in phenolic content at higher substitution levels reflects the contribution of mocaf and pumpkin flour to the bioactive profile of the wet noodles. Moreover, this enhancement contributes to the improved functional properties of wet noodles as a source of bioactive compounds.

The antioxidant activity of wet noodles increased from 2.69% to 49.75% (*p* < 0.05). This increase reflects a synergistic effect between *β*‐carotene and phenolic compounds in scavenging free radicals [49]. Carotenoid compounds, such as *β*‐carotene, are known to act as free radical scavengers via electron or hydrogen‐atom donation mechanisms, thereby playing a key role in suppressing oxidation in food systems [50]. The antioxidant activity of phenolic compounds is attributed to their capacity to donate protons and stabilize free radicals [51]. The interaction between these two groups of bioactive compounds can produce a synergistic effect, enhancing the overall antioxidant capacity of the fresh noodle product. The observed increase in antioxidant activity is consistent with the higher *β*‐carotene and TPCs observed in formulations containing greater proportions of pumpkin flour and mocaf. The *β*‐carotene content of pumpkin, along with the phenolic compounds present in the raw materials, contributes to the product′s increased antioxidant potential [10]. Overall, the increase in these bioactive components indicates that the incorporation of mocaf and pumpkin flour enhanced the functional properties of the wet noodles through increased *β*‐carotene content, TPC, and antioxidant activity. The use of local ingredients in noodle formulations facilitates food diversification and simultaneously contributes to the development of functional food products with potential health benefits.

### 3.5. Starch Digestibility and eGI

Starch digestibility fractions, including RDS, SDS, RS, and the eGI of fresh noodles prepared with varying proportions of wheat flour, mocaf, and pumpkin flour, are summarized in Table 5.

**Table 5:** Starch digestibility fractions and estimated glycemic index of wet noodles.

Wheat flour: mocaf: pumpkin flour (*w*/*w*)	RDS (%)	SDS (%)	RS (%)	eGI
100:0:0	64.69 ± 2.97^a^	20.73 ± 1.02^e^	5.76 ± 0.18^e^	73.87 ± 1.90^a^
80:10:10	60.75 ± 2.04^b^	22.42 ± 0.97^d^	7.65 ± 0.95^d^	70.93 ± 2.07^b^
70:20:10	57.86 ± 1.97^c^	24.65 ± 1.53^c^	8.71 ± 0.87^c^	67.86 ± 1.86^c^
60:20:20	52.74 ± 1.86^d^	26.86 ± 1.91^b^	10.15 ± 0.42^b^	63.74 ± 1.63^d^
50:25:25	49.08 ± 1.04^e^	28.94 ± 1.74^a^	12.62 ± 0.86^a^	58.06 ± 1.07^e^

*Note:* Data are reported as mean ± SD, and statistical differences within a column are indicated by distinct superscript letters (*p* < 0.05).

As presented in Table 5, increasing the proportions of mocaf and pumpkin flour significantly influenced the starch digestibility fractions and the eGI of fresh noodles (*p* < 0.05). The RDS value decreased gradually from 64.69% in the control formulation (100:0:0) to 49.08% in the formulation with the highest substitution (50:25:25). The reduction in the RDS fraction suggests that a portion of the starch becomes less susceptible to enzymatic hydrolysis during the initial phase of digestion [52]. Fiber and pectin may act as a physical barrier that limits the access of amylase enzymes to starch granules, thereby reducing the rate of starch hydrolysis [53]. In addition, interactions between starch and nonstarch components, such as fiber and phenolic compounds, can result in a more compact and stable matrix structure, ultimately reducing enzyme access to the starch substrate. This denser matrix structure can also limit the diffusion of water and enzymes during in vitro digestion, thereby reducing the fraction of RDS [54]. The reduction in RDS and the corresponding increase in SDS and RS may also be associated with starch–fiber interactions within the noodle matrix. Dietary fiber and pectin derived from pumpkin flour can form a physical barrier surrounding starch granules, thereby restricting enzyme accessibility during digestion. Furthermore, hydrogen bonding between starch molecules and nonstarch polysaccharides may contribute to the formation of a more compact matrix structure, resulting in slower starch hydrolysis and a lower estimated glycemic response.

The SDS value increased from 20.73% to 28.94%, whereas the RS value increased from 5.76% to 12.62% (*p* < 0.05). The observed changes indicate that substitution with mocaf and pumpkin flour influences the digestibility profile of starch in fresh noodles. Furthermore, the inclusion of pumpkin flour has been shown to enhance nutritional attributes, particularly by increasing dietary fiber and lowering glycemic response, supporting its application as a functional substitute ingredient [55, 56]. A decrease in the RDS fraction indicates that some of the starch has become less susceptible to hydrolysis by digestive enzymes [57]. Interactions between starch and other macromolecular components can enhance matrix compactness and stability, resulting in reduced enzyme penetration and slower starch hydrolysis [58].

[29] The increase in SDS and RS fractions suggests that starch hydrolysis occurred more gradually during digestion. RS is less susceptible to enzymatic degradation and may exert physiological effects similar to dietary fiber by escaping digestion in the small intestine and undergoing fermentation in the colon [59]. Therefore, the increased RS content may contribute to the enhanced functional properties of the developed fresh noodles.

Changes in the starch fraction were also reflected in the eGI values of the products. The estimated GI value decreased significantly from 73.87 in the control formulation to 58.06 in the formulation with the highest substitution. The decrease in GI value indicates that increasing the proportion of mocaf and pumpkin flour could yield noodles with a lower glycemic response. Changes in starch fractions, characterized by lower RDS and higher SDS and RS, are closely linked to the observed decrease in glycemic index [60]. Foods containing more SDS fractions tend to release glucose at a slower rate, resulting in a lower glycemic response [61].

The results indicate that alterations in starch fractions are consistently linked to corresponding changes in the eGI of fresh noodle products. A decrease in the RDS fraction, accompanied by increases in SDS and RS, contributes to a decrease in the eGI value [62]. The functional value of noodle products can be improved through the use of mocaf and pumpkin flour, as this combination alters starch composition, reduces glycemic response, and increases the fraction of SDS. It should be noted that the glycemic index values reported in this study were estimated from an in vitro digestion model and may differ from glycemic responses observed in vivo due to physiological factors affecting digestion, absorption, and glucose metabolism.

### 3.6. Spectrum FTIR

**Figure 2:**
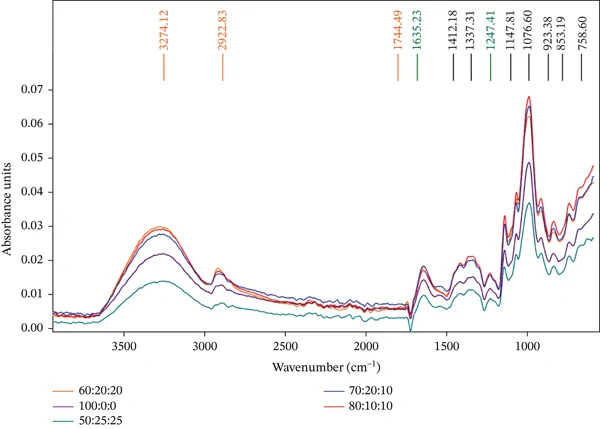
FTIR spectra of wet noodles formulated with different wheat flour: mocaf: pumpkin flour ratios.

Figure 2 illustrates the FTIR spectra obtained from wet noodle formulations containing different proportions of wheat flour, mocaf, and pumpkin flour. All formulations exhibit relatively similar spectral patterns, indicating that the main components of the wet noodles remain dominated by carbohydrates and proteins. The absorption band at approximately 3274 cm^−1^ is associated with the stretching of hydroxyl (O–H) groups from water molecules, starch, and dietary fiber [63]. The band at approximately 2920 cm^−1^ is associated with the stretching of the C–H bond in the organic components of the material matrix [64]. The intensity of this band was higher in formulations with a higher proportion of mocaf and pumpkin flour, such as in the 60:20:20 and 50:25:25 ratios, suggesting a possible increase in hydrogen bonding within the matrix.

The absorption band observed around 2922 cm^−1^ corresponds to C–H stretching vibrations of organic constituents. The peak at around 1653 cm^−1^ corresponds to the amide I region of wheat proteins, which are important for noodle matrix formation. Additionally, the fingerprint region (1200–900 cm^−1^) exhibits strong bands at 1076, 1022, and 995 cm^−1^, representing C–O and C–O–C vibrations of starch polysaccharides [65]. Interactions between starch, protein, and nonstarch components of mocaf and pumpkin flour are indicated by changes in band intensity in this region at higher substitution levels [66]. These interactions can modify starch molecular structure and affect its functional behavior and digestibility in the final noodle product.

### 3.7. Microstructure

**Figure 3:**
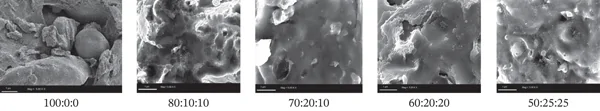
SEM micrographs of wet noodles formulated with different wheat flour: mocaf: pumpkin flour ratios.

Microstructural alterations in wet noodle formulations containing varying proportions of wheat flour, mocaf, and pumpkin flour were examined by SEM, as presented in Figure 3. In the control formulation (100:0:0), the noodle structure exhibited a relatively compact and continuous matrix with starch granules trapped within the gluten network.

This structure reflects the ability of wheat flour to form a cohesive protein network, resulting in a more uniform dough matrix. However, increasing the proportions of mocaf and pumpkin flour led to a more irregular microstructure, characterized by the presence of pores and surface gaps. This effect is attributed to the reduction in gluten content caused by partial substitution with nonwheat ingredients. In addition, higher levels of fiber and other nonstarch components from mocaf and pumpkin flour may disrupt the formation of a continuous gluten network within the dough matrix [67]. In the 60:20:20 formulation, the microstructure still exhibits a relatively homogeneous matrix compared with formulations with higher substitution levels, indicating a better balance between starch and protein components in the dough system. The more open microstructure in formulations with higher substitution levels can enhance water diffusion during the cooking process and improve enzyme accessibility during in vitro starch digestion [68]. These conditions can affect the textural characteristics and digestibility fractions of the starch in the resulting fresh noodles.

### 3.8. Sensory Evaluation

**Figure 4:**
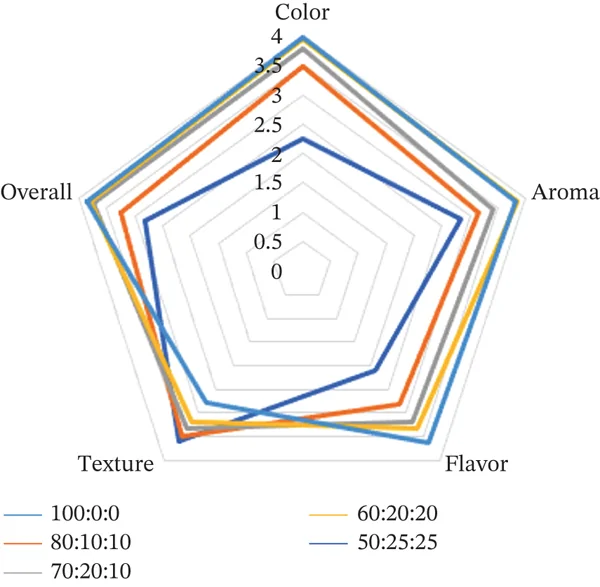
Sensory acceptance profile of wet noodles formulated with different wheat flour: mocaf: pumpkin flour ratios.

Figure 4 demonstrates that variations in the proportions of wheat flour, mocaf, and pumpkin flour significantly affected sensory attributes, including color, aroma, taste, texture, and overall acceptance. Color preference scores increased with the addition of pumpkin flour up to a 60:20:20 formulation. The observed yellow–orange hue results from the presence of carotenoid pigments, notably *β*‐carotene, which enhance the visual attractiveness of the product to panelists. Carotenoids are known to give a characteristic yellow–orange color to pumpkin‐based products [36, 49].

The aroma score of the wet noodles also increased to a moderate level of substitution. The slightly sweet and distinctive aroma of pumpkin flour was considered to add character to the product. However, in the 50:25:25 formulation, there was a decrease in the aroma score, indicating that an excessively high proportion of pumpkin flour resulted in a stronger and less desirable sweet aroma. The aroma of wet noodles originates from pumpkin, which has a pleasant scent reminiscent of fresh fruit [69].

The flavor attributes of wet noodles show a similar trend. Adding moderate amounts of pumpkin flour can enhance the perception of natural sweetness and give the product a soft texture. This flavor is dominated by the addition of pumpkin flour. Pumpkin has a distinctive fresh fruit flavor [70]. The 60:20:20 formulation received the highest score because the panelists considered the balance of the flour flavor, the natural sweetness of the pumpkin flour, and the distinctive aroma to be in just the right proportions. The highest formulation (50:25:25) actually lowered the flavor score due to the overly strong pumpkin flavor.

The texture value of wet noodles decreased in the 50:25:25 formulation, in line with instrumental texture data showing a decrease in hardness and an increase in softness. Panelists preferred the texture of the 60:20:20 formulation, which exhibited moderate elasticity, a smoother surface, and a structure that remained fairly compact. This combination was consistent with the results of texture analysis and SEM, which showed that the noodle structure remained intact [35].

Overall, the 60:20:20 formulation had the highest likability score among the formulations. This formulation exhibited a more appealing color, a balanced aroma and flavor, and a texture that most closely matched the characteristics of fresh noodles expected by the panelists. The combination of wheat flour, mocaf, and pumpkin flour in that ratio produced fresh noodles with good sensory quality and high acceptance among the panelists. The sensory characteristics of the final product are strongly influenced by the balance of ingredient composition. Substituting wheat flour with mocaf and pumpkin flour at moderate levels improves color and sensory attributes while maintaining acceptable texture quality [71]. Conversely, an excessively high substitution rate can affect the starch and protein matrix structure in the dough, potentially reducing the noodles′ elasticity and chewiness. Therefore, the 60:20:20 formulation can be considered the most optimal for producing fresh noodles with balanced sensory characteristics that are well‐received by consumers.

## 4. Conclusions

Partial substitution of wheat flour with mocaf and pumpkin flour significantly affected the physicochemical properties, bioactive compounds, starch digestibility, and structural characteristics of wet noodles. Increasing substitution levels reduced texture and deformation while increasing cooking loss due to the weakening of the gluten network and modifications in the starch–protein matrix. The incorporation of mocaf and pumpkin flour increased *β*‐carotene content, total phenolic compounds, and antioxidant activity, while promoting a more favorable starch digestibility profile. Structural observations using FTIR and SEM confirmed changes in the molecular and microstructural properties of the noodle matrix. The 60:20:20 formulation provided the best balance between structural integrity and sensory acceptance. Overall, these findings demonstrate that mocaf and pumpkin flour can be effectively utilized to develop functional wet noodles with improved nutritional and functional properties. These findings support the potential industrial application of mocaf and pumpkin flour as locally available ingredients for the development of value‐added functional noodle products. Future studies should evaluate in vivo glycemic responses, storage stability, and consumer acceptance under commercial processing conditions.

## Author Contributions

Agus Slamet: conceptualization, methodology, formal analysis, investigation, data curation, writing—original draft preparation, supervision, funding acquisition, and correspondence. Wafit Dinarto: methodology, investigation, data curation, and writing—review and editing. Sundari Sundari: supervision, resources, and writing—review and editing.

## Funding

This study was supported by Directorate of Research and Community Service, Directorate General of Research and Development, Ministry of Higher Education, Science and Technology, Republic of Indonesia, SP DIPA‐139.04.1.693320/2025.

## Ethics Statement

The sensory evaluation in this study was approved by the Ethics Committee of Universitas Alma‐Ata, Yogyakarta, Indonesia (Reference No.: KE/AA/X/10112097/EC/2025).

## Conflicts of Interest

The authors declare no conflicts of interest.

## Data Availability

The data supporting the findings of this study are available within the article. Additional data are available from the corresponding author upon reasonable request.

## References

[1] Knorr D. and Augustin M. A. , Expanding our Food Supply: Underutilized Resources and Resilient Processing Technologies, Journal of the Science of Food and Agriculture. (2025) 105, no. 2, 735–746, 10.1002/jsfa.13740, 38989972. 38989972 PMC11632173

[2] Mahmud N. , Islam J. , and Tahergorabi R. , Noodles, Cereal-Based Food Products, 2023, Springer International Publishing, 221–252, 10.1007/978-3-031-40308-8_10.

[3] Kapsdorferová Z. , Bogueva D. , and Marinova D. , Consumer Attitudes and Views on Sustainable Food Consumption, Consumer Perceptions and Food, 2024, Springer Nature Singapore, 299–317, 10.1007/978-981-97-7870-6_15.

[4] Zhang M. , Jia R. , Ma M. , Yang T. , Sun Q. , and Li M. , Versatile Wheat Gluten: Functional Properties and Application in the Food-Related Industry, Critical Reviews in Food Science and Nutrition. (2023) 63, no. 30, 10444–10460, 10.1080/10408398.2022.2078785, 35608010. 35608010

[5] Shobur M. , Nyoman Marayasa I. , Bastuti S. , Muslim A. C. , Pratama G. A. , and Alfatiyah R. , Enhancing Food Security Through Import Volume Optimization and Supply Chain Communication Models: A Case Study of East Java′s Rice Sector, Journal of Open Innovation: Technology, Market, and Complexity. (2025) 11, no. 1, 100462, 10.1016/j.joitmc.2024.100462.

[6] Roncaglia F. , Ughetti A. , Severini A. , Porcelli N. , Mazzali M. , D′Eusanio V. , and Rigamonti L. , Biofuel Production From Nature′s Sugars: New Horizons in the Carbohydrate Economy, Sustainable Energy Technologies and Assessments. (2026) 86, 104880, 10.1016/j.seta.2026.104880.

[7] Matita I. C. , Soedirga L. C. , and Andriani I. , Utilization of Chia Seeds Powder in Wet Noodle Substituted With Modified Cassava Flour, Caraka Tani: Journal of Sustainable Agriculture. (2023) 39, no. 1, 10.20961/carakatani.v39i1.77711.

[8] Zaidiyah Z. , Abubakar A. , Indarti E. , and Anwar S. H. , Analysis of Spontaneous and Biological Fermented Cassava Flour for Sourdough Application, IOP Conference Series: Earth and Environmental Science. (2024) 1290, no. 1, 012051, 10.1088/1755-1315/1290/1/012051.

[9] Soedirga L. C. and Kitesvara R. A. , Dry Noodle Made from Modified Cassava and Red Beet Composite Flour With Various Concentrations of Xanthan Gum, Food Research. (2025) 9, no. 5, 10.26656/fr.2017.9(5).143.

[10] Slamet A. , Kanetro B. , and Purwaningsih H. , Physicochemical Properties, Antioxidant Activities, Beta-Carotene Content, and Sensory Properties of Gluten-Free Pumpkin-Fortified Egg Roll, Food Research. (2026) 10, no. 1, 10.26656/fr.2017.10(1).047.

[11] Gomes R. S. , Machado Júnior R. , de Almeida C. F. , de Oliveira R. L. , Nascimento M. , Nardino M. , do Nascimento W. F. , and da Silva D. J. H. , Artificial Neural Networks Optimize the Establishment of a Brazilian Germplasm Core Collection of Winter Squash (Cucurbita moschata D), Science Reports. (2024) 14, no. 1, 5930, 10.1038/s41598-024-54818-y, 38467669. PMC10928206 38467669

[12] Setiawan B. , Aulia S. S. , Sulaeman A. , Kusharto C. M. , and Handharyani E. , Isoflavone and Antioxidant of Instant Cream Soup Made From Pumpkin and Tempeh and Their Active Compound in Ovariohysterectomy Rat-Induced Alzheimer′s Disease, International Journal of Food Science. (2022) 2022, 7, 10.1155/2022/8051624. PMC9722274 36479443

[13] Ma D. , Han P. , Song M. , Zhang H. , Shen W. , Huang G. , Zhao M. , Sun Q. , Zhao Y. , and Min L. , *β*-Carotene Rescues Busulfan Disrupted Spermatogenesis Through Elevation in Testicular Antioxidant Capability, Frontiers in Pharmacology. (2021) 12, 593953, 10.3389/fphar.2021.593953, 33658940. 33658940 PMC7917239

[14] Stryjecka M. , Krochmal-Marczak B. , Cebulak T. , and Kiełtyka-Dadasiewicz A. , Assessment of Phenolic Acid Content and Antioxidant Properties of the Pulp of Five Pumpkin Species Cultivated in Southeastern Poland, International Journal of Molecular Sciences. (2023) 24, no. 10, 10.3390/ijms24108621, 37239966. PMC10218659 37239966

[15] Slamet A. , Kanetro B. , and Setiyoko A. , The Hypoglycemic and Regenerative Effect of the Pancreas Using Instant Porridge Mix of Pumpkin and Brown Rice Flour on Diabetic Rats, Potravinarstvo Slovak Journal of Food Sciences. (2022) 16, 92–100, 10.5219/1705.

[16] Lerdluksamee C. , Thanaboonrongkom S. , Dajanta K. , Pongjanta J. , Chaikham P. , and Jirasatid S. , Techno-Functional, Nutritional, and Sensory Properties of Pumpkin Flour-Enriched Dried Egg Noodles, Applied Food Research. (2025) 5, no. 2, 101399, 10.1016/j.afres.2025.101399.

[17] Raghunathan R. , Farahnaky A. , Majzoobi M. , Chandrapala J. , Brennan C. , Eri R. , Rustagi S. , and Pandiselvam R. , Slowly Digestible Starch: A Biochemical Engineering Perspective on Functional Food Development and Metabolic Health, Journal of Food Measurement and Characterization. (2025) 19, no. 8, 5197–5221, 10.1007/s11694-025-03322-6.

[18] Bello-Perez L. A. and Hoyos-Leyva J. D. , Development of Foods High in Slowly Digestible and Resistant Starch, Starch in Food, 2018, Elsevier, 827–854, 10.1016/B978-0-08-100868-3.00022-6.

[19] Doan H. T. T. , Kim T. , Cha M. , and Kim S.-J. , Synthesis and Potential Application of Slowly Digestible Starch, Journal of Functional Foods. (2025) 131, 106955, 10.1016/j.jff.2025.106955.

[20] Chen R. , Zhu Y. , Sang L. , Zhao L. , Sharafeldin S. , Zhi L. , Wu C. , Zhao Q. , and Shen Q. , Relationship Between Gluten Structural Properties and Noodle Texture: Insights From Seven Wheat Varieties, Food Bioengineering. (2025) 4, no. 2, 210–221, 10.1002/fbe2.70011.

[21] Ding C. , Cai W.-H. , Sun J.-Y. , Tao H. , and Wang H.-L. , Unveiling the Binding Mechanism Between Starch Granule-Surface Proteins and Glutenins During Dough Mixing Process, Food Hydrocolloids. (2024) 150, 109760, 10.1016/j.foodhyd.2024.109760.

[22] An H. , Ma Q. , Zhang F. , Zhai C. , Sun J. , Tang Y. , and Wang W. , Insight Into Microstructure Evolution During Starch Retrogradation by Infrared and Raman Spectroscopy Combined With Two-Dimensional Correlation Spectroscopy Analysis, Food Hydrocolloids. (2024) 146, 109174, 10.1016/j.foodhyd.2023.109174.

[23] He R. , Li S. , Zhao G. , Zhai L. , Qin P. , and Yang L. , Starch Modification With Molecular Transformation, Physicochemical Characteristics, and Industrial Usability: A State-of-the-Art Review, Polymers. (2023) 15, no. 13, 10.3390/polym15132935. PMC10346181 37447580

[24] AOAC , Official Standard of Analysis of OAC International, 2005, 16th edition, AOAC International. Arlington, Virginia, USA: Association of Official Analytical Chemists, Inc.

[25] Li J. , Qi Y. , Ahmed Z. , and Xu B. , A Methodological Study of the Hardness of Cooked Chinese Noodles Based on the Texture Analyzer, International Journal of Food Engineering. (2024) 20, no. 7, 551–560, 10.1515/ijfe-2024-0049.

[26] Mohammed H. H. , Tola Y. B. , Taye A. H. , and Abdisa Z. K. , Effect of Pretreatments and Solar Tunnel Dryer Zones on Functional Properties, Proximate Composition, and Bioactive Components of Pumpkin (Cucurbita maxima) Pulp Powder, Heliyon. (2022) 8, no. 10, e10747, 10.1016/j.heliyon.2022.e10747, 36203905, e10747. 36203905 PMC9529547

[27] Singleton V. L. , Orthofer R. , and Lamuela-Raventós R. M. , [14] Analysis of Total Phenols and Other Oxidation Substrates and Antioxidants by Means of Folin-Ciocalteu Reagent, Methods in Enzymology, 1999, 299, 152–178, 10.1016/S0076-6879(99)99017-1.

[28] Brand-Williams W. , Cuvelier M. E. , and Berset C. , Use of a Free Radical Method to Evaluate Antioxidant Activity, LWT-Food Science and Technology. (1995) 28, no. 1, 25–30, 10.1016/S0023-6438(95)80008-5.

[29] Li W. , Zhang W. , Gong S. , Gu X. , Yu Y. , Wu J. , and Wang Z. , Low and High Methoxyl Pectin Lowers on Structural Change and Digestibility of Fried Potato Starch, LWT. (2020) 132, 109853, 10.1016/j.lwt.2020.109853.

[30] Goñi I. , Garcia-Alonso A. , and Saura-Calixto F. , A Starch Hydrolysis Procedure to Estimate Glycemic Index, Nutrition Research. (1997) 17, no. 3, 427–437, 10.1016/S0271-5317(97)00010-9.

[31] Huang L. , Zhao J. , Wei Y. , Yu G. , and Li Q. , Characterization of a Neutral Polysaccharide From Pumpkin (Cucurbita moschata Duch) With Potential Immunomodulatory Activity, International Journal of Biological Macromolecules. (2021) 188, 729–739, 10.1016/j.ijbiomac.2021.08.053, 34389393. 34389393

[32] Slamet A. , Praseptiangga D. , Hartanto R. , and Samanhudi , Moisture Sorption Isotherm and Shelf Life of Pumpkin and Arrowroot Starch-Based Instant Porridge, AIP Conference Proceedings, 2020, American Institute of Physics Inc., 10.1063/5.0003102.

[33] Kulczynski B. and Gramza-Michałowska A. , The Profile of Carotenoids and Other Bioactive Molecules in Various Pumpkin Fruits (Cucurbita maxima Duchesne) Cultivars, Molecules. (2019) 24, no. 18, 10.3390/molecules24183212, 31487816. PMC6766813 31487816

[34] Aji G. K. , Budiyanto , Laksono H. , Wiguna B. , Putri R. P. G. , Kusumasmarawati A. D. , Muhamaludin , Mufti A. , Rutmala A. , Rosyidin A. K. , and Nasori A. S. , Property Evaluation of Noodles Substituting Wheat Flour With Drum-Dried Overripe Kepok Plantain (*Musa paradisiaca* L.) Flour to Enhance the Nutrients, Preventive Nutrition and Food Science. (2024) 29, no. 4, 554–562, 10.3746/pnf.2024.29.4.554, 39759818. 39759818 PMC11699572

[35] Nanthachai N. , Lichanporn I. , Tanganurat P. , and Kumnongphai P. , Development of Pumpkin Powder Incorporated Instant Noodles, Current Research in Nutrition and Food Science. (2020) 8, no. 2, 524–530, 10.12944/CRNFSJ.8.2.18.

[36] Slamet A. , Kanetro B. , and Purwaningsih H. , Physicochemical and Sensory Evaluation of Pumpkin-Based Instant Porridge With Mocaf and Cowpea Flour, Scifood. (2025) 19, 96–109, 10.5219/scifood.8.

[37] Too B. C. , Van Tai N. , and Thuy N. M. , Formulation and Quality Evaluation of Noodles With Starchy Flours Containing High Levels of Resistant Starch, Acta Scientiarum. Technology. (2022) 21, no. 2, 145–154, 10.17306/J.AFS.2022.1011.

[38] Ihedinachi O. A. , Udeh C. C. , Emojorho E. E. , Amonyeze A. O. , Nwaorgu S. I. , and Aniemena C. C. , Evaluation of Nutritional Qualities of Complementary Food Produce From Malted Rice, Soybean and Pumpkin Pulp Flour, Food Chemistry Advances. (2025) 6, 100863, 10.1016/j.focha.2024.100863, 100863.

[39] Ouyang M. , Huang Y. , Wang Y. , Luo F. , and Liao L. , Stability of Carotenoids and Carotenoid Esters in Pumpkin (Cucurbita maxima) Slices During Hot Air Drying, Food Chemistry. (2022) 367, 130710, 10.1016/j.foodchem.2021.130710, 130710, 34343802. 34343802

[40] Shang Y. , Jiang T. , Liu Y. , Xu Y. , Gao Y. , Xiao H. , Ma Y. , Yang S. , and Wei Z. , Effect of Phytochemicals From Grape Seed on the Improvement of Noodle Quality and the Structure of Wheat Gluten, LWT. (2025) 218, 117482, 10.1016/j.lwt.2025.117482.

[41] Mostafa S. , Ata S. M. , Hussein A. M. S. , and Zaky A. A. , Development and Quality Evaluation of High-Protein Gluten-Free Pasta Formulations, Science Reports. (2025) 15, no. 1, 10.1038/s41598-025-12336-5, 40715416. PMC12297300 40715416

[42] Tan H. L. , Yong K. C. , Tan T. C. , and Mat Easa A. , Impact of Various Tea Infusions on the Cooking, Texture, and Microstructure of Fresh White Salted Noodles, International Journal of Gastronomy and Food Science. (2025) 39, 101089, 10.1016/j.ijgfs.2024.101089, 101089.

[43] Zhang Q. , Chen M. , Li W. , Liang C. , Huang X. , Hu H. , Huang Z. , Gan T. , and Zhang Y. , Effects of the Addition of Cassava Starch and the Size of Water Clusters on Physicochemical and Cooking Properties of Rice Noodles, Food Chemistry. (2025) 470, 142665, 10.1016/j.foodchem.2024.142665, 142665, 39733622. 39733622

[44] Ninčević Grassino A. , Rimac Brnčić S. , Badanjak Sabolović M. , Šic Žlabur J. , Marović R. , and Brnčić M. , Carotenoid Content and Profiles of Pumpkin Products and By-Products, Molecules. (2023) 28, no. 2, 10.3390/molecules28020858, 36677916. PMC9861221 36677916

[45] Beer K. , Singh A. , Ravi S. C. , Gupta A. K. , Kumar A. , and Sharma M. M. , A Comprehensive Review on the Role of Vitamin A on Human Health and Nutrition, Journal of Environmental Biology. (2024) 45, no. 6, 645–653, 10.22438/jeb/45/6/MRN-5339.

[46] Pruthvi G. , Apoorva M. , Anuthilakesh T. , Bhargavi K. , Nithish G. S. , and Achar R. , Food Polyphenols, Polyphenols, 2023, 1–20, 10.1002/9781394188864.ch1.

[47] Matić M. , Stupar A. , Kebert M. , Kiprovski B. , and Brdar-Jokanović M. , The Role of Carotenoids in Preventing Oxidative Stress: Exploring the Antioxidant Potential of Pumpkin as a Functional Crop, Food and Feed Research. (2026) 53, no. 1, 79–91, 10.5937/ffr0-56562.

[48] Zhang Q. , Cheng Z. , Wang Y. , and Fu L. , Dietary Protein-Phenolic Interactions: Characterization, Biochemical-Physiological Consequences, and Potential Food Applications, Critical Reviews in Food Science and Nutrition. (2021) 61, no. 21, 3589–3615, 10.1080/10408398.2020.1803199, 32814438. 32814438

[49] Indrianingsih A. W. , Rosyida V. T. , Darsih C. , Apriyana W. , Iwansyah A. C. , Khasanah Y. , Kusumaningrum A. , Windarsih A. , Herawati E. R. N. , Muzdalifah D. , and Sulistyowaty M. I. , Physicochemical Properties, Antioxidant Activities, *Β*-Carotene Content, and Sensory Properties of Cookies From Pumpkin (Cucurbita moschata) and Modified Cassava Flour (Manihot esculenta), Bioactive Carbohydrates and Dietary Fibre. (2024) 31, 100398, 10.1016/j.bcdf.2023.100398, 100398.

[50] Yildiz Z. I. , Topuz F. , Kilic M. E. , Durgun E. , and Uyar T. , Encapsulation of Antioxidant Beta-Carotene by Cyclodextrin Complex Electrospun Nanofibers: Solubilization and Stabilization of Beta-Carotene by Cyclodextrins, Food Chemistry. (2023) 423, 136284, 10.1016/j.foodchem.2023.136284, 37156137. 37156137

[51] Zeb A. , Concept, Mechanism, and Applications of Phenolic Antioxidants in Foods, Journal Food Biochemistry. (2020) 44, no. 9, 10.1111/jfbc.13394, 32691460. 32691460

[52] Xu M. , Suo T. , Chen Y. , Guo L. , Zhang Z. , Li C. , Wang Z. , and Zou J. , Dynamic Structural Changes of Chinese Yam Starch-Konjac Glucomannan Complexes During Hydrolysis: Molecular Determinants for Time-Dependent Digestive Modulation, Food Research International. (2026) 223, no. 1, 117857, 10.1016/j.foodres.2025.117857, 41352795. 41352795

[53] Chen R. , Williams P. A. , Shu J. , Luo S. , Chen J. , and Liu C. , Pectin Adsorption Onto and Penetration Into Starch Granules and the Effect on the Gelatinization Process and Rheological Properties, Food Hydrocolloid. (2022) 129, 107618, 10.1016/j.foodhyd.2022.107618.

[54] Zhao J. , Shen M. , Wang R. , Gu Z. , Hong Y. , Li Z. , Li C. , Ban X. , and Cheng L. , Effects of Removing Endogenous Non-Starch Components on the Structure, Physicochemical Properties, and In Vitro Digestibility of Castanopsis Sclerophylla Flour, Food Research International. (2026) 230, 118567, 10.1016/j.foodres.2026.118567, 41794454. 41794454

[55] Wang Y. , Tang N. , Geng D.-H. , Wang K. , Asiamah E. , and Cheng Y. , Understanding the Processing and Digestibility Attributes of Rice Noodles Supplemented With Acetylated Cassava Starch, International Journal of Biological Macromolecules. (2025) 315, no. 2, 144393, 10.1016/j.ijbiomac.2025.144393, 40398780. 40398780

[56] Alija D. , Olędzki R. , Nikolovska Nedelkoska D. , Wojciechowicz-Budzisz A. , Xhabiri G. , Pejcz E. , Alija E. , and Harasym J. , The Addition of Pumpkin Flour Impacts the Functional and Bioactive Properties of Soft Wheat Composite Flour Blends, Foods. (2025) 14, no. 2, 10.3390/foods14020243, 39856909. PMC11764949 39856909

[57] Jo M. , Qi J. , Du Z. , Li Y. , and Shi Y.-C. , Changes in the Structure and Enzyme Binding of Starches During In Vitro Enzymatic Hydrolysis Using Mammalian Mucosal Enzyme Mixtures, Carbohydrate Polymers. (2024) 335, 122070, 10.1016/j.carbpol.2024.122070, 38616092. 38616092

[58] Chen X. , Zhu L. , Zhang H. , Wu G. , Cheng L. , and Zhang Y. , A Review of Endogenous Non-Starch Components in Cereal Matrix: Spatial Distribution and Mechanisms for Inhibiting Starch Digestion, Critical Reviews in Food Science and Nutrition. (2025) 65, no. 19, 3686–3701, 10.1080/10408398.2024.2370487. 38920118

[59] Ahmmed R. , Paff A. , Kong L. , Li S. , Cockburn D. W. , and Tan L. , Effect of Resistant Starch Type 5 on Gut Health Through Modulating Gut Microbiota, Engineering Microbiology. (2026) 6, no. 1, 100250, 10.1016/j.engmic.2025.100250, 41982390. 41982390 PMC13064624

[60] Thuy N. M. , Too B. C. , Vuong K. M. , Lan P. T. , Tuyen P. T. , Tram N. B. , and Van Tai N. , Resistant Starch in Various Starchy Vegetables and the Relationship With Its Physical and Chemical Characteristics,, Journal of Applied Biology and Biotechnology. (2022) 10, no. 1, 181–188, 10.7324/JABB.2021.100122.

[61] Wang Y. , Zhou X. , Xiang X. , and Miao M. , Association of Slowly Digestible Starch Intake With Reduction of Postprandial Glycemic Response: An Update Meta-Analysis, Foods. (2022) 12, no. 1, 10.3390/foods12010089. PMC9818736 36613304

[62] Jiang X. , Zheng F. , Yu J. , Lv P. , Ban H. , Liu H. , Cai D. , Xiu L. , and Liu J. , Effects of Static Magnetic Field Treatment on the Digestive, Structural and Physicochemical Characteristics of Germinated Corn Starch, Food Chemistry. (2025) 470, 142670, 10.1016/j.foodchem.2024.142670, 39733621. 39733621

[63] Lazarev S. I. , Golovin Y. M. , Khorokhorina I. V. , and Khokhlov P. A. , A Study of the Structural Organization of the Surface Layer and the State of Water in Composite Ultrafiltration Membranes, Protection of Metals and Physical Chemistry of Surfaces. (2020) 56, no. 2, 262–267, 10.1134/S207020512002015X.

[64] Bakri M. K. , Rahman M. R. , Hamdan S. , Nyuk Khui P. L. , Jayamani E. , and Kakar A. , Infrared Spectral Functional Group and Thermal Properties of Acacia Wood Bio-Composites, Acacia Wood Bio-Composites: Towards Bio-Sustainability of the Environment, 2019, 135–151, 10.1007/978-3-030-29627-8_6.

[65] Srivastava Y. , Chandwani N. , Dave P. K. , and Patil C. , Comparative Structural and Techno-Functional Properties of Barnyard, Browntop and Little Millet Starch by Dielectric Gas Discharge and Vacuum Plasma, Vegetos. (2025) 10.1007/s42535-025-01315-w.

[66] Xie J. , Cheng L. , Li Z. , Li C. , Hong Y. , and Gu Z. , Effect of Non-Starch Components on the Structural Properties, Physicochemical Properties and In Vitro Digestibility of Waxy Highland Barley Starch, International Journal of Biological Macromolecules. (2024) 255, 128013, 10.1016/j.ijbiomac.2023.128013, 37951447. 37951447

[67] Hu X. , Cheng L. , Hong Y. , Li Z. , Li C. , and Gu Z. , Impact of Celluloses and Pectins Restrictions on Gluten Development and Water Distribution in Potato-Wheat Flour Dough, International Journal of Biological Macromolecules. (2022) 206, 534–542, 10.1016/j.ijbiomac.2022.02.150, 35235853. 35235853

[68] Contardo I. , James B. , and Bouchon P. , Microstructural Characterization of Vacuum-Fried Matrices and Their Influence on Starch Digestion, Food Structure. (2020) 25, 100146, 10.1016/j.foostr.2020.100146.

[69] Provesi J. G. , Dias C. O. , and Amante E. R. , Changes in Carotenoids During Processing and Storage of Pumpkin Puree, Food Chemistry. (2011) 128, no. 1, 195–202, 10.1016/j.foodchem.2011.03.027, 25214348. 25214348

[70] Rasdi M. R. , Zainal Abidin M. , and Malik N. H. , Effect of Drying Conditions on Functional Properties of Pumpkin Powder, Advances in Agricultural and Food Research Journal. (2024) 5, no. 1, 10.36877/aafrj.a0000481.

[71] Aljahani A. H. and Al-Khuarie A. N. , Effect of Mixing Wheat Flour With Pumpkin and Dates on the Nutritional and Sensory Characteristics of Cake, Pakistan Journal of Nutrition. (2017) 16, no. 4, 273–278, 10.3923/pjn.2017.273.278.

